# Supplementary material for: Genome-Wide DNA Methylation Patterns of Muscle and Tail-Fat in DairyMeade Sheep and Mongolian Sheep
Source: Animals (Basel). 2022 May 29;12(11):1399. doi: 10.3390/ani12111399 (PMC9179529; doi:10.3390/ani12111399)
Supplement: Supplementary file 1 [file animals-12-01399-s001.zip › animals-1685280-supplementary/animals-1685280-supplementary Figures.pdf]

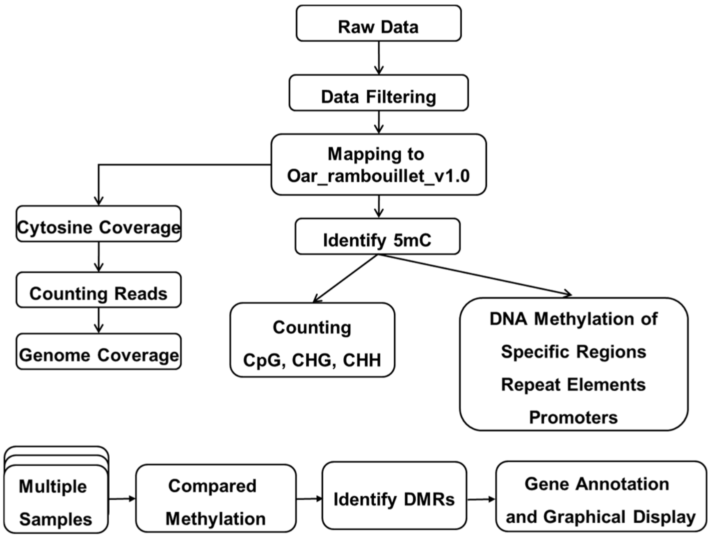

Figure S1. The flow chart showed the data analysis of Whole-Genome Bisulfite Sequencing.

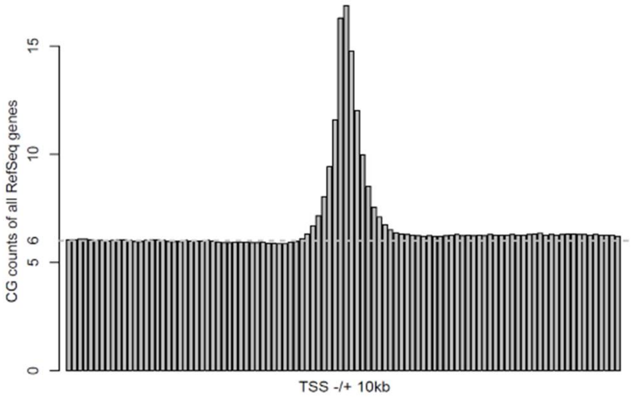

Figure S2. The distribution of CpG counts of the 100 bins (TSS ± 10kb) for all the RefSeq genes.
